# PP2A inhibitor SET promotes mTORC1 and Bmi1 signaling through Akt activation and maintains the colony-formation ability of cancer cells

**DOI:** 10.1016/j.jbc.2023.105584

**Published:** 2023-12-22

**Authors:** Naoki Kohyanagi, Nao Kitamura, Shunta Ikeda, Shusaku Shibutani, Koichi Sato, Takashi Ohama

**Affiliations:** 1 Laboratory of Veterinary Pharmacology, Yamaguchi University Joint Graduate School of Veterinary Medicine, Yamaguchi, Japan; 2 Laboratory of Veterinary Hygiene, Yamaguchi University Joint Graduate School of Veterinary Medicine, Yamaguchi, Japan

**Keywords:** Akt/PKB, Bmi-1, cancer biology, mammalian target of rapamycin, polycomb, protein phosphatase 2A, SET

## Abstract

Protein phosphatase 2A (PP2A) is an essential tumor suppressor, with its activity often hindered in cancer cells by endogenous PP2A inhibitory proteins like SE translocation (SET). SET/PP2A axis plays a pivotal role in the colony-formation ability of cancer cells and the stabilization of c-Myc and E2F1 proteins implicated in this process. However, in osteosarcoma cell line HOS, SET knock-down (KD) suppresses the colony-formation ability without affecting c-Myc and E2F1. This study aimed to unravel the molecular mechanism through which SET enhances the colony-formation ability of HOS cells and determine if it is generalized to other cancer cells. Transcriptome analysis unveiled that SET KD suppressed mTORC1 signaling. SET KD inhibited Akt phosphorylation, an upstream kinase for mTORC1. PP2A inhibitor blocked SET KD-mediated decrease in phosphorylation of Akt and a mTORC1 substrate p70S6K. A constitutively active Akt restored decreased colony-formation ability by SET KD, indicating the SET/PP2A/Akt/mTORC1 axis. Additionally, enrichment analysis highlighted that Bmi-1, a polycomb group protein, is affected by SET KD. SET KD decreased Bmi-1 protein by Akt inhibition but not by mTORC1 inhibition, and exogenous Bmi-1 expression rescued the reduced colony formation by SET KD. Four out of eight cancer cell lines exhibited decreased Bmi-1 by SET KD. Further analysis of these cell lines revealed that Myc activity plays a role in SET KD-mediated Bmi-1 degradation. These findings provide new insights into the molecular mechanism of SET-regulated colony-formation ability, which involved Akt-mediated activation of mTORC1/p70S6K and Bmi-1 signaling.

Excessive protein phosphorylation because of abnormal activation of kinases and inactivation of phosphatases contribute to cancer development and malignancy. Protein phosphatase 2A (PP2A) is a pivotal intracellular serine (Ser)/threonine (Thr) protein phosphatase and an essential tumor suppressor (1). The decrease in PP2A activity has been observed in various cancers, and the increase in endogenous PP2A inhibitory proteins such as SE translocation (SET, also called I2PP2A and TAF-1) contributes to this. Increased SET protein has been observed in a variety of cancers, including acute myeloid leukemia, gastric cancer, breast cancer, pancreatic cancer, prostate cancer, colorectal cancer, non–small cell lung cancer, and hepatocellular carcinoma, with a positive correlation between high SET expression and poor prognosis (2, 3, 4, 5, 6, 7, 8, 9).

Accumulating evidence revealed molecular mechanisms of SET-mediated cancer promotion, and the involvement of c-Myc protein stabilization is widely accepted (5, 6, 10). PP2A dephosphorylates c-Myc to induce proteasomal degradation, and the elevated SET inhibits this pathway. On the contrary, our previous report showed that SET knockdown (KD) suppresses the colony-formation ability of all human cancer cell lines tested, but a decrease in c-Myc protein is observed in only some cell lines (4). Stabilization of E2F1 was revealed as another molecular mechanism for cancer promotion by the SET–PP2A axis. However, some cell lines, including the osteosarcoma cell line HOS, showed reduced colony-formation ability without affecting both c-Myc and E2F1 proteins. These results suggest a third molecular mechanism other than c-Myc and E2F1. Akt is a well-known substrate of PP2A, and SET KD reduces the phosphorylation level of Akt in several cancer cell lines (5, 11, 12). However, Akt is an upstream factor of c-Myc and E2F1, and it is not clear how SET-induced Akt activation benefits cancer cells without affecting protein levels of c-Myc and E2F1. In the present study, transcriptome analysis of HOS cells revealed the involvement of mammalian target of rapamycin complex 1 (mTORC1) and B-lymphoma Moloney murine leukemia virus insertion region-1 (Bmi-1).

mTOR is the Ser–Thr kinase that plays critical roles in many aspects of cellular function in physiological and pathological conditions (13). The mTOR forms complexes with several proteins to yield holoenzymes termed mTORC1 and mTORC2. mTORC1 is the downstream factor of Akt and phosphorylates 4EBP1 and p70S6 kinase (S6K) to initiate ribosomal translation of mRNA required for cell growth and metabolism. Bmi-1 forms polycomb repressive complex 1 (PRC1) with Ring1B and other proteins, which functions as a ubiquitin ligase for histone H2A and induces H2A monoubiquitination to lock chromatin in a silenced state (14). Elevated levels of Bmi-1 protein have been detected in various cancer types, including osteosarcoma (15, 16, 17). Bmi-1 promotes cancer stemness not only as a subunit of PRC1 but also in a PRC1-independent manner (18, 19, 20, 21, 22). Akt signaling induces Bmi-1 phosphorylation and protein stabilization (23, 24). Our data added new insights into the involvement of Akt-mediated activation of mTORC1–p70S6K and Bmi-1 signaling in the SET-regulated colony-formation ability.

## Results

### SET maintains the colony-formation ability of HOS cells by a mechanism independent of c-myc and E2F1 degradation

In our previous work (4), HOS and SW620 cells showed that SET KD reduced colony-formation ability without affecting both c-Myc and E2F1 proteins. We confirmed that the suppression of SET to 90% in HOS cells did not alter c-Myc and E2F1 protein (Fig. S1, *A* and *B*), whereas SET KD reduced the colony-formation ability of HOS cells (Fig. S1, *C* and *D*). On the other hand, a decrease in c-Myc expression was observed in SW620 cells when SET protein levels were reduced by more than 90% and colony-formation ability was reduced by about 40% (Fig. S1, *E*–*H*). The phosphorylation levels of Ser62 and the half-life of c-Myc were not altered by SET KD, whereas MYC mRNA expression was lower in SET KD SW620 cells (Fig. S1, *I*–*M*), suggesting the transcriptional regulation. The discrepancies with a previous report may be because SET expression was suppressed by only about 50% in the past report. Therefore, we employed HOS cells to elucidate a novel molecular mechanism by which SET maintains the colony-formation ability of cancer cells.

**Figure 1:**
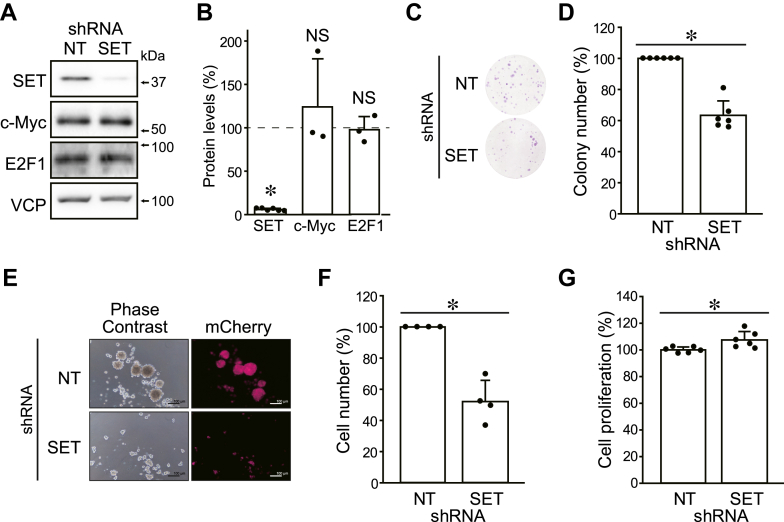
**SET maintains the colony-formation ability of HOS cells by a mechanism independent of c-Myc and E2F1 degradation.** HOS cells were expressed shNT and shSET by DOX treatment. *A* and *B*, protein levels were analyzed by immunoblotting. Representative images (*A*) and quantitative data (*B*) are shown. The band densities of shSET-expressing cells were normalized to those of shNT-expressing cells at 100%. *C* and *D*, a colony-formation assay. Representative images (*C*) and quantitative data (*D*) are shown. The number of colonies of shSET was normalized to those of shNT at 100%. *E* and *F*, a spheroid formation assay. Representative images (*E*) and quantitative data (*F*) are shown. The number of cells of shSET was normalized to that of shNT at 100%. *G*, cell proliferation was analyzed using Cell Counting Kit 8. The proliferation rate of shSET was normalized to that of shNT at 100%. ∗*p* < 0.05. NS, not significantly different. Data points are independent biological replicates. DOX, doxycycline; shNT, nontarget shRNA; shSET, SET-targeting shRNA.

For this purpose, we generated HOS cells expressing SET-targeting shRNA (shSET) in a doxycycline (DOX)-inducible manner. DOX treatment decreased SET protein by about 90% (Fig. 1, *A* and *B*). Similar to the stable SET KD, inducible SET KD did not affect c-Myc and E2F1 protein levels in HOS cells (Fig. 1, *A* and *B*). DOX treatment decreased colony-formation ability, whereas no difference was observed in the DOX-untreated cells (Figs. 1, *C* and *D* and S1, *N* and *O*). A similar phenomenon was observed for another shSET (shSET #2) (Fig. S1, *P*–*S*). We also found that DOX-induced SET KD suppressed anchorage-independent growth but did not suppress cell proliferation (Fig. 1, *E*–*G*). These data suggest that SET maintains the stemness of HOS cells *via* an unidentified molecular mechanism.

### SET activates the Akt–mTORC1 pathway by inhibiting PP2A

**Figure 2:**
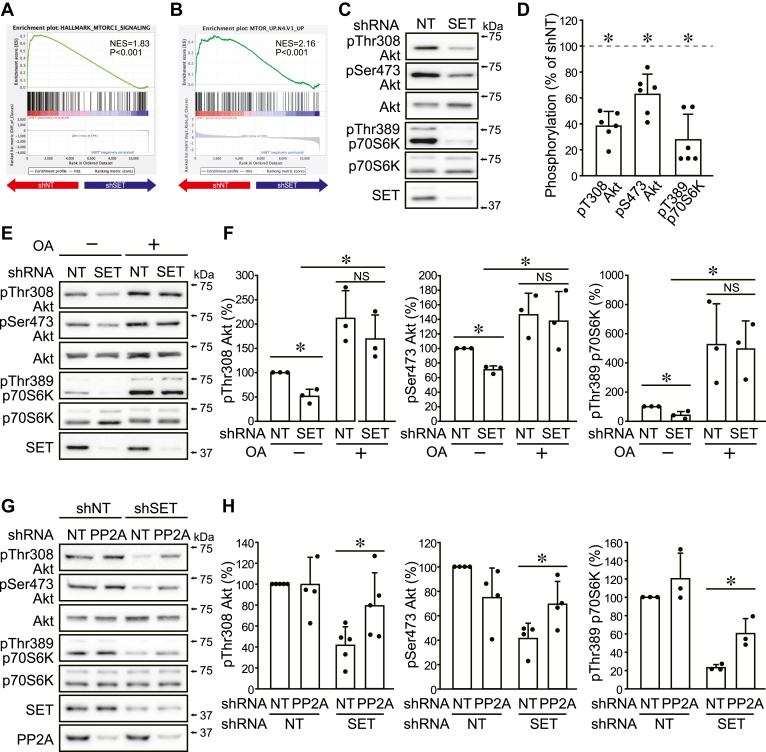
**SET activates the Akt–mTORC1 pathway by inhibiting PP2A.***A*–*F*, HOS cells were expressed shNT and shSET by DOX treatment. *A* and *B*, gene set enrichment analysis for the transcriptome data of shNT- and shSET-expressing cells. Enrichment plots for (*A*) HALLMARK_MTORC1_SIGNALING and (*B*) MTOR_UP.N4.V1_UP are shown. *C* and *D*, immunoblotting was performed for indicated antibodies. Representative images (*C*) and quantitative data (*D*) are shown. The band densities were normalized to shNT at 100%. *E* and *F*, HOS cells expressing shNT or shSET were cultured with or without okadaic acid (OA; 100 nM) for 2 h, and immunoblotting was performed for indicated antibodies. Representative images (*E*) and quantitative data (*F*) are shown. The band densities were normalized to shNT/OA− at 100%. *G* and *H*, HOS cells were expressed shNT and shPP2A, and then shNT and shSET were expressed by DOX treatment. Immunoblotting was performed for indicated antibodies. Representative images (*G*) and quantitative data (*H*) are shown. The band densities were normalized to shNT/shNT at 100%. ∗*p* < 0.05. NS, not significantly different. Data points are independent biological replicates. DOX, doxycycline; mTORC1, mammalian target of rapamycin complex 1; PP2A, protein phosphatase 2A; SET, SE translocation; shNT, nontarget shRNA; shSET, SET-targeting shRNA.

To comprehensively understand the molecular mechanism affected by SET KD, transcriptome analysis was performed. Among the HALLMARK gene sets of gene set enrichment analysis (GSEA), the top five gene sets that were significantly enriched (nominal [NOM] *p* < 0.05 and false discovery rate *q* < 0.25) in the nontarget shRNA (shNT)- and shSET-expressing cells are shown in Fig. S2*A*. Since SET KD did not alter c-Myc and E2F1 protein level, we focused on mTORC1 signaling that was also indicated in the C6 oncogenic signature collection (Figs. 2, *A* and *B* and S2, *A* and *B*). Immunoblotting confirmed that SET KD decreased the p70S6K phosphorylation at Thr389, an indicator of mTORC1 activation (Fig. 2, *C* and *D*). We also found that SET KD suppressed the Thr308 and Ser473 phosphorylation of Akt, an upstream kinase of mTORC1 and a substrate of PP2A (Fig. 2, *C* and *D*). A similar phenomenon was observed for shSET #2-expressing cells (Fig. S2, *C* and *D*). On the contrary, SET KD did not alter the phosphorylation levels of 4EBP1 at Thr37/46, another substrate of mTORC1 (Fig. S2, *E* and *F*). Since 4EBP1, in contrast to p70S6K, is resistant to mTOR inhibitor rapamycin (25), the decrease in mTORC1 activity by SET KD may be limited as is rapamycin. The phosphorylation levels of 3-phosphoinositide-dependent protein kinase-1, an upstream kinase of Akt, and the colocalization of mTOR and lysosome, an indicator of mTOR activation *via* amino acids, were not affected by SET KD (Fig. S2, *E*–*G*), suggesting SET targets Akt to promote mTORC1–p70S6K pathway. SET KD–mediated decrease in Akt and p70S6K phosphorylation was rescued by the PP2A inhibitor okadaic acid (OA) and PP2A KD (Figs. 2, *E*–*H* and S2*H*). These results indicate that SET activates the Akt–mTORC1 pathway by inhibiting PP2A in HOS cells.

### The Akt–mTORC1 pathway is involved in the SET-mediated stemness

**Figure 3:**
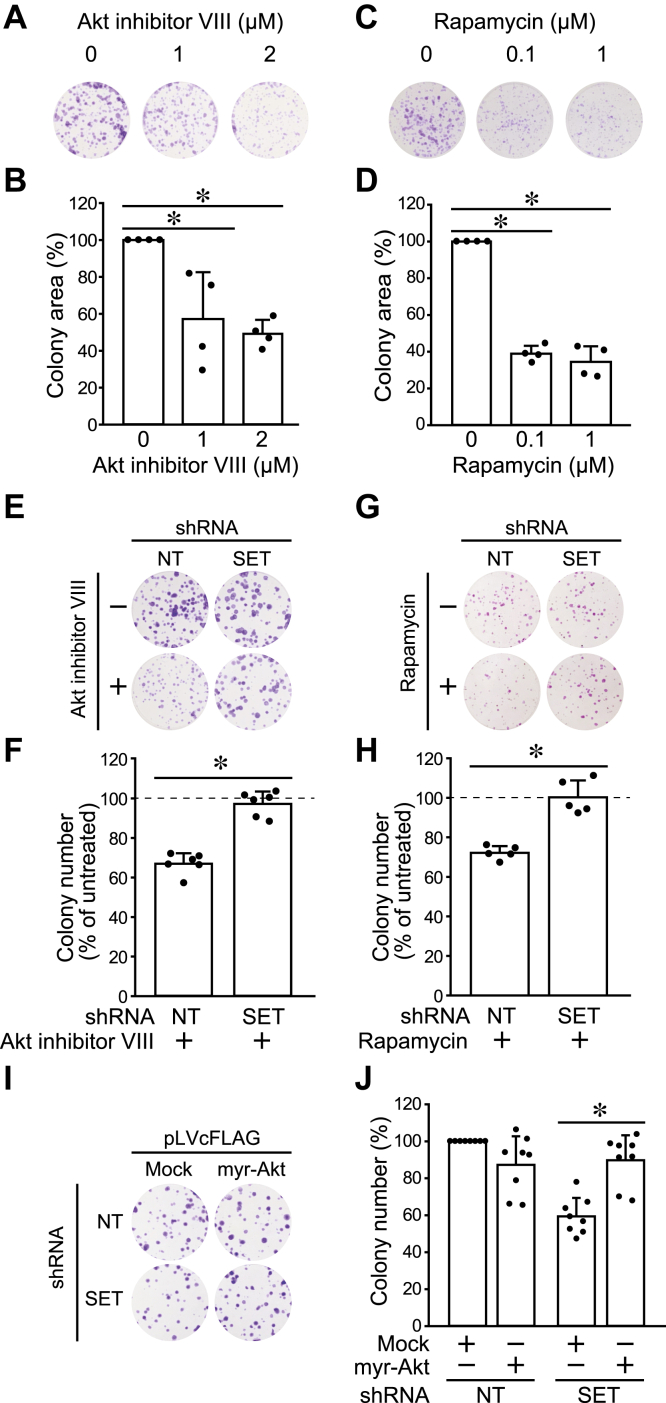
**The Akt–mTORC1 pathway is involved in the SET-mediated stemness.***A*–*D*, the effects of Akt inhibitor VIII (*A* and *B*) and rapamycin (*C* and *D*) on the colony-formation ability of HOS cells were analyzed. Representative pictures (*A* and *C*) and quantitative data (*B* and *D*) are shown. The total area of colonies was normalized to untreated cells at 100%. *E*–*J*, HOS cells were expressed shNT and shSET by DOX treatment. *E*–*H*, the effects of Akt inhibitor VIII (2 μM) (*E* and *F*) and rapamycin (1 μM) (*G* and *H*) on the colony-formation ability were analyzed. Representative pictures (*E* and *G*) and quantitative data (*F* and *H*) are shown. The number of colonies was normalized to untreated cells at 100%. *I* and *J*, the effects of myr-Akt expression on the colony-formation ability were analyzed. Representative pictures (*I*) and quantitative data (*J*) are shown. The number of colonies was normalized to shNT/mock at 100%. ∗*p* < 0.05. Data points are independent biological replicates. DOX, doxycycline; mTORC1, mammalian target of rapamycin complex 1; myr-Akt, myristrated Akt; SET, SE translocation; shNT, nontarget shRNA; shSET, SET-targeting shRNA.

Treatment of HOS cells with Akt inhibitor (Akt inhibitor VIII) and mTOR inhibitor (rapamycin and Torin1) markedly reduced colony-formation ability (Figs. 3, *A*–*D* and S3, *A*–*E*), indicating that the Akt–mTORC1 pathway is involved in colony formation of HOS cells. These compounds did not reduce colony formation of HOS cells expressing SET-targeting shRNA, probably because Akt–mTOR pathway was already suppressed by SET KD (Fig. 3, *E*–*H*).

Myristrated Akt (myr-Akt) is the constitutively active form of Akt (26). Expression of myr-Akt induced p70S6K phosphorylation in HOS cells expressing shSET to the same level as in shNT-expressing HOS cells (Fig. S3*F*). We found that myr-Akt expression rescued decreased colony and spheroid formations by SET KD (Figs. 3, *I* and *J* and S3, *G* and *H*). These results suggested that the Akt–mTORC1 pathway plays a pivotal role in the SET-mediated colony-formation ability in HOS cells.

### SET stabilizes Bmi-1 through Akt activation

**Figure 4:**
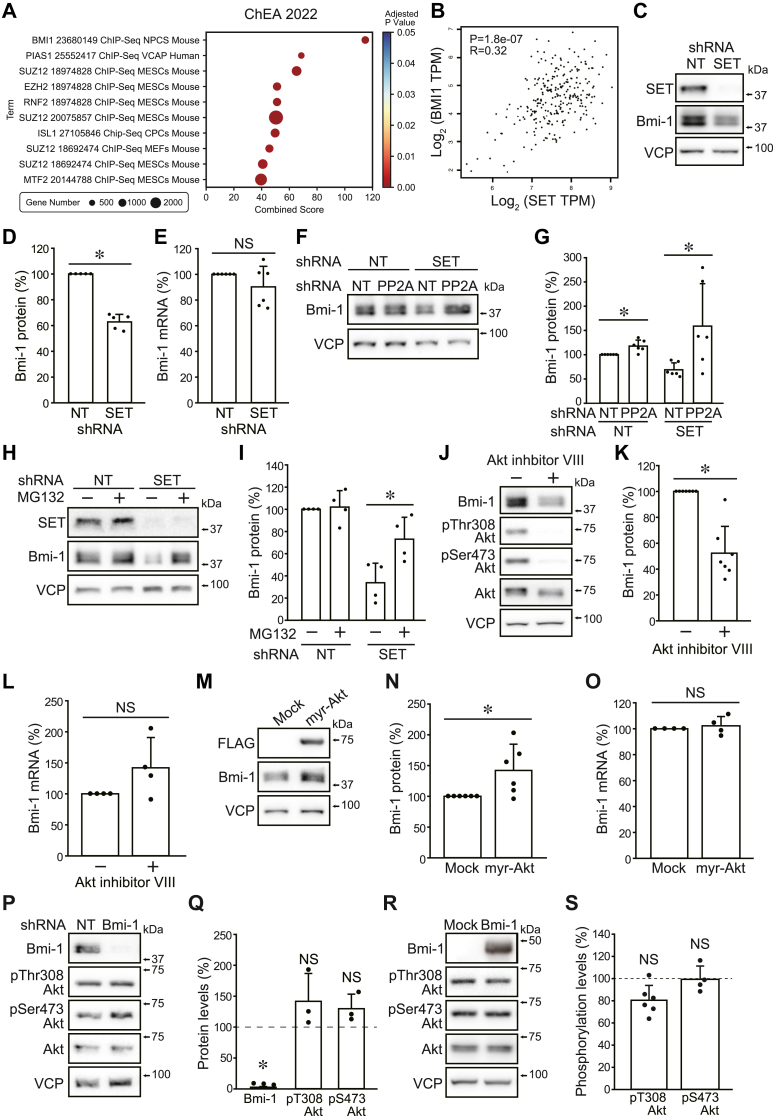
**SET stabilizes Bmi-1 through Akt activation.***A*, enrichment analysis for the ChEA_2022 gene set library. The top 10 enriched terms for the DEGs are displayed based on the combined score. *B*, correlation analysis between *SET* and *BMI1* expressions in SARC (sarcoma) dataset using GEPIA. *C*–*I*, HOS cells were expressed shNT and shSET by DOX treatment. *C* and *D*, immunoblotting was performed for indicated antibodies. The band densities were normalized to shNT at 100%. *E*, real-time PCR. The relative value of *BMI1* mRNA expression was normalized to shNT at 100%. *F* and *G*, the effect of PP2A KD on SET KD–induced Bmi-1 reduction was analyzed. The band densities were normalized to shNT/shNT at 100%. *H* and *I*, cells were treated with MG132 (100 nM) for 4 h, and immunoblotting was performed for indicated antibodies. The band densities were normalized to shNT/MG132(−) at 100%. *J*–*O*, the effects of Akt inhibitor VIII (10 μM, 24 h) (*J*–*L*) and myr-Akt expression (*M*–*O*) on protein (*J*, *K*, *M* and *N*) and mRNA (*L* and *O*) expressions were analyzed. The band densities and *BMI1* mRNA expressions were normalized to untreated or mock-expressing cells at 100%. *P*–*S*, proteins were extracted from HOS cells expressing shNT and shBmi-1 (*P* and *Q*) and mock and FLAG-Bmi-1 (*R* and *S*). Immunoblotting was performed for indicated antibodies. Representative images (*P* and *R*) and quantitative data (*Q* and *S*) are shown. The band densities were normalized to those of shNT or mock-expressing cells at 100%. ∗*p* < 0.05. NS, not significantly different. Data points are independent biological replicates. Bmi-1, B-lymphoma Moloney murine leukemia virus insertion region-1; DEG, differentially expressed gene; DOX, doxycycline; KD, knockdown; myr-Akt, myristrated Akt; PP2A, protein phosphatase 2A; SET, SE translocation; shNT, nontarget shRNA; shSET, SET-targeting shRNA.

Enrichment analysis with Enrichr (https://maayanlab.cloud/Enrichr/) was performed using 83 differentially expressed genes decreased by SET KD (Fig. S4*A*). Enrichr showed that genes identified by chromatin immunoprecipitation sequencing of anti-Bmi-1 antibodies were enriched (Fig. 4*A*). Moreover, correlation analysis using GEPIA (http://gepia.cancer-pku.cn/index.html) showed a positive correlation between *SET* and *BMI1* expressions in SARC (sarcoma) dataset (Fig. 4*B*). Since Akt phosphorylates Bmi-1 to inhibit its ubiquitin-proteasomal degradation (25, 26), we hypothesized that Bmi-1 stabilization by Akt may contribute to SET-mediated colony-formation ability. Consistent with this hypothesis, SET KD reduced the Bmi-1 protein level without altering mRNA expression (Figs. 4, *C*–*E* and S4, *B* and *C*). SET-KD–induced reduction in Bmi-1 protein was blocked by PP2A KD (Fig. 4, *F* and *G*). Treatment cells with OA showed slow migrating phosphorylated Bmi-1 bands suggesting that PP2A inhibition leads to Bmi-1 phosphorylation (Fig. S4*D*). Proteasome inhibitor MG132 rescued SET KD–induced Bmi-1 degradation (Fig. 4, *H* and *I*). Akt inhibitor VIII also decreased Bmi-1 protein but did not affect Bmi-1 mRNA expression (Figs. 4, *J*–*L* and S4, *E* and *F*). On the contrary, activation of Akt signaling by myr-Akt increased Bmi-1 proteins, whereas it did not affect Bmi-1 mRNA expression (Fig. 4, *M*–*O*). SET KD and Akt inhibitor also decreased Ring1B protein levels (Fig. S4, *G*–*J*), suggesting SET may maintain the PRC1 complex *via* Akt activation. Rapamycin did not alter Bmi-1 protein in shNT-expressing cells but rescued Bmi-1 suppression by SET KD to some extent, probably because of Akt activation by a feedback mechanism (Fig. S4, *K*–*O*). Bmi-1-targeting shRNA almost completely suppressed Bmi-1 expression but did not suppress Akt phosphorylation (Fig. 4, *P* and *Q*). Moreover, Bmi-1 overexpression did not increase Akt phosphorylation (Fig. 4, *R* and *S*). These data suggest that Akt is located upstream of Bmi-1, causing Bmi-1 protein stabilization in an mTORC1-independent manner.

### Bmi-1 stabilization is involved in SET-mediated colony-formation ability

**Figure 5:**
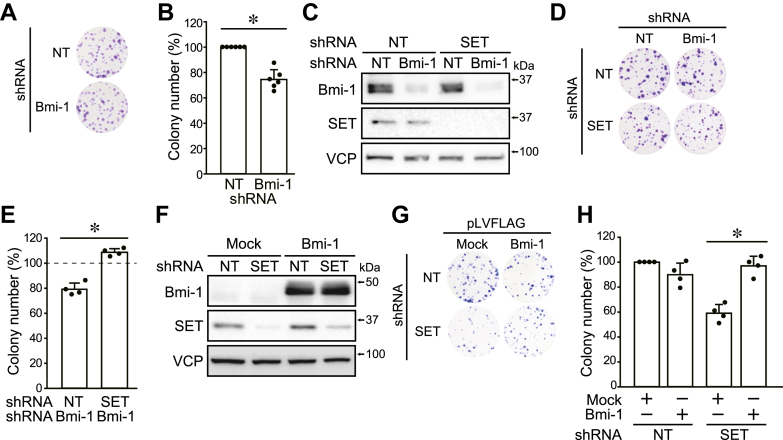
**Bmi-1 stabilization is involved in SET-mediated colony-formation ability.***A* and *B*, the effects of Bmi-1 KD on the colony-formation ability of HOS cells were analyzed. Representative pictures (*A*) and quantitative data (*B*) are shown. The number of colonies was normalized to shNT-expressing cells at 100%. *C*–*H*, HOS cells were expressed shNT and shSET by DOX treatment. The effect of Bmi-1 KD and Bmi-1 overexpression on colony-formation ability was analyzed. Immunoblotting was performed for the indicated antibodies (*C* and *F*). Representative pictures (*D* and *G*) and quantitative data (*E* and *H*) are shown. The number of colonies was normalized to shNT-expressing or shNT/mock-expressing cells at 100%. ∗*p* < 0.05. Data points are independent biological replicates. Bmi-1, B-lymphoma Moloney murine leukemia virus insertion region-1; DOX, doxycycline; KD, knockdown; SET, SE translocation; shNT, nontarget shRNA; shSET, SET-targeting shRNA.

To confirm the involvement of Bmi-1 in SET-mediated stemness, we used Bmi-1 inhibitor PTC-209, Bmi-1-targeting shRNA, and Bmi-1 expression plasmids. Bmi-1 KD decreased colony formation to about 70% (Fig. 5, *A* and *B*). PTC-209 decreased Bmi-1 protein levels to about 60% and reduced colony formation to about 80% (Fig. S5, *A*–*D*). Bmi-1 KD and PTC-209 decreased the colony formation of shNT-expressing cells but did not affect shSET-expressing cells (Figs. 5, *C*–*E* and S5, *E* and *F*). Moreover, exogenous expression of Bmi-1 rescued SET KD–induced reduction in colony- and spheroid-formation abilities (Figs. 5, *F*–*H* and S5, *G* and *H*). These results suggest that Bmi-1 stabilization by Akt is involved in the SET-mediated colony-formation ability.

### Myc activity plays a pivotal role in SET KD–mediated Bmi-1 degradation

**Figure 6:**
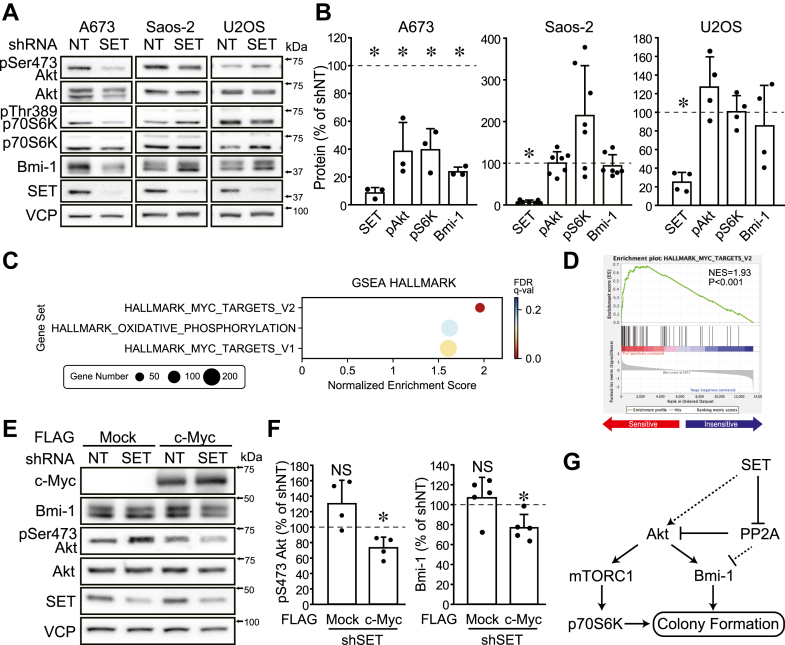
**Myc activity plays a pivotal role in SET KD–mediated Bmi-1 degradation.***A* and *B*, SET expression was suppressed in bone cancer cell line, and immunoblotting was performed for indicated antibodies. Representative images (*A*) and quantitative data (*B*) are shown. The band densities were normalized to shNT at 100%. C and *D*, gene set enrichment analysis for the transcriptome data of cell lines sensitive and insensitive for SET KD–mediated Bmi-1 degradation. *C*, significantly enriched gene sets in GSEA HALLMARK. *D*, enrichment plots for MYC_TARGETS_V2. *E* and *F*, the combined effects of SET KD and c-Myc overexpression in Bmi-1 protein levels were analyzed in 293T cells. Representative images (*E*) and quantitative data (*F*) are shown. ∗*p* < 0.05. NS, not significantly different. *G*, a proposed model of this study. *Solid lines* indicate pathways identified in this study, and *dotted lines* indicate possible pathways. Data points are independent biological replicates. Bmi-1, B-lymphoma Moloney murine leukemia virus insertion region-1; GSEA, gene set enrichment analysis; KD, knockdown; SET, SE translocation; shNT, nontarget shRNA.

To determine whether the SET KD–induced Bmi-1 degradation is an HOS cell–specific phenomenon, SET protein was decreased in other bone cancer (osteosarcoma and Ewing sarcoma) cell lines. SET KD decreased phosphorylation levels of Akt and p70S6K and protein levels of Bmi-1 in A673 cells (Fig. 6, *A* and *B*). On the other hand, SET KD did not suppress these factors in Saos-2 and U2OS cells (Fig. 6, *A* and *B*).

The bone cancer groups that are sensitive and insensitive to SET KD–induced Bmi-1 degradation showed no correlation regarding Bmi-1, SET, PP2A, and Akt phosphorylation levels (Fig. S6, *A* and *B*). We further analyzed the effects of SET KD on Bmi-1 protein in MIA PaCa-2 (pancreatic cancer), A549 (lung cancer), HT-29 (colon cancer), and SH-SY5Y (neuroblastoma). SET KD decreased Bmi-1 protein in MIA PaCa-2 and A549 cells but not in HT-29 and SH-SY5Y cells (Fig. S6, *C* and *D*). To note, SET KD suppressed Akt activity but did not decrease Bmi-1 protein in HT-29 cells. The exogenous expression of Bmi-1 and myr-Akt rescued decreased colony and spheroid formations of A549 cells, supporting the results of HOS cells (Fig. S6, *E*–*N*).

To estimate the molecular mechanism that produces the difference, we obtained transcriptome data of these cell lines from the depmap portal (https://depmap.org/portal/) (Fig. S7*A*) and performed GSEA between sensitive (HOS, A673, A549, and MIA PaCa-2) and insensitive (Saos-2, U2OS, SH-SY5Y, and HT-29) for SET KD–mediated Bmi-1 degradation. Among the HALLMARK gene sets, three and zero gene sets were significantly enriched (NOM *p* < 0.05 and false discovery rate *q* < 0.25) in the sensitive and insensitive groups, respectively. Two of the three gene sets were related to Myc, indicating that the sensitive group has higher Myc activity (Figs. 6, *C* and *D* and S7*B*). In 293T cells, SET KD did not alter Bmi-1 protein, but the combination of exogenous c-Myc and SET KD decreased Bmi-1 protein (Figs. 6, *E* and *F* and S7*C*). These data suggest that Myc activity is one of the key factors for Bmi-1 degradation by SET KD.

## Discussion

Increased SET protein plays a pivotal role in the progression of various cancer types. Accumulating evidence revealed that the degradations of c-Myc and E2F1 through PP2A inactivation are the dominant mechanisms of SET-mediated cancer cell stemness. However, our previous report found that these two proteins cannot explain all molecular mechanisms (4). In this study, we revealed that the SET-mediated Akt activation promotes mTOR–p70S6K and Bmi-1 signaling. Although this study primarily used HOS cells, Bmi-1 protein was decreased by SET KD in multiple cell lines, and our data suggest that Myc activity is one of the pivotal factors for this phenotype.

The PP2A inhibitor OA and PP2A KD almost blocked the SET KD–induced decreased phosphorylation levels of Akt and mTORC1 substrate p70S6K, suggesting that SET activates Akt–mTORC1–p70S6K by inhibiting PP2A. SET KD and SET inhibitor OP449 suppressed mTORC1 signaling in canine osteosarcoma and human breast cancer cell lines (27, 28). Interestingly, SET-induced activation of mTORC1 signaling was limited to p70S6K and did not affect 4EBP1. Because 4EBP1 is resistant to mTORC1 inhibitor rapamycin (25), the decrease in mTORC1 activity by SET KD may be limited as is rapamycin. Rapamycin associates with the FK506-binding protein 12, which binds to a domain adjacent to the active site of mTOR and blocks the access of substrates to the active site, whereas torin1 binds to the ATP-binding site of mTOR (29). The limited effect of rapamycin was also evident in its action on HOS cell colony formation. The effect of rapamycin reached a plateau, whereas torin1 completely inhibited colony formation. This is consistent with the limited reduction in HOS cell colony formation by SET KD. The reason for the limitation of mTORC1 inhibition by SET remains to be elucidated.

SET KD–induced reduction of Bmi-1 protein was blocked by PP2A KD, suggesting the involvement of PP2A in Bmi-1 degradation. Since HOS cells floated out after 4 h of OA treatment, Bmi-1 accumulation was not observed with OA. However, 2 h treatment with OA shifted the Bmi-1 band upward, presumably because of the phosphorylation of Bmi-1. These data suggest that PP2A regulates the Bmi-1 phosphorylation levels. There are no reports of PP2A directly dephosphorylating Bmi-1, and the possibility for an indirect effect *via* kinase activation cannot be excluded. Our data showed that the activation of Akt signaling resulted in Bmi-1 protein accumulation. Consistent with our study, in neural stem cells, activation of Akt signaling results in Bmi-1 phosphorylation and protein stabilization (24). However, it is not clear which sites of Bmi-1 are phosphorylated by Akt. Casein kinase 2 stabilizes Bmi-1 by phosphorylating Ser110 (30). Akt may phosphorylate this site, but the sequence around Ser110 differs from the consensus phosphorylation site of Akt. Ser251, 253, and 255 of Bmi-1 are implicated as potential phosphorylation sites by Akt, and phosphorylation of these sites enhances the oncogenic potential of Bmi-1 in mouse prostate cancer (23). However, the increase in Bmi-1 protein was not observed in that report. Moreover, Bmi-1 inhibitor PTC-596 decreases Bmi-1 protein but induces its phosphorylation (31). The relationship between Bmi-1 phosphorylation and proteolysis has not been established.

Bmi-1 is involved in the maintenance of stemness in normal and cancer cells. As a subunit of PRC1, Bmi-1 represses the transcription of the *CDKN2A*, which encodes the cell cycle regulator p16 (Ink4a) and the tumor suppressor p19 (Arf), to promote self-renewal of stem cells (21, 32, 33, 34). Liu *et al.* (35) reported that Akt phosphorylates Ser316 of Bmi-1 to block its transcriptional repressor activity toward the *Ink4a–Arf1* locus, suggesting that Akt-mediated Bmi-1 phosphorylation suppresses stemness. The discrepancy with our results can be explained by the fact that HOS cells harbor the deletion in the *CDKN2A* gene (c.1_471del471). To note, all the cell lines sensitive for SET KD–mediated Bmi-1 degradation (HOS, A673, A549, and MIA PaCa-2) harbor *CDKN2A* c.1_471del471, whereas the insensitive group (Saos-2, U2OS, HT-29, and SH-SY5Y) has wildtype *CDKN2A*. A larger number of cell lines need to be analyzed to determine whether the *CDKN2A* products are involved in the SET-mediated stabilization of the Bmi-1 protein.

In HT-29 cells, Bmi-1 expression was not decreased despite suppression of Akt activity by SET KD. These data suggest that the suppression of Akt activity is not enough to induce Bmi-1 degradation. Transcriptome analysis of public data suggested that the sensitive group has higher Myc activity and exogenous c-Myc-sensitized cells for SET KD–induced Bmi-1 reduction. These data indicate that c-Myc is one of the pivotal factors for this phenotype. Bmi-1 was first identified as a protein that cooperatively promotes tumorigenesis with c-Myc in B-cell lymphoma (36). Various factors were reported to be involved in Bmi-1 protein stabilization–degradation. Ubiquitin-specific protease 15, ubiquitin-specific protease 22, basic transcription factor 3, and O-linked *N*-acetylglucosamine transferase stabilize Bmi-1, whereas βTrCP promotes Bmi-1 degradation (37, 38, 39, 40). There were no differences in transcriptome analysis between sensitive and insensitive groups for any of these factors. Since Myc is a master regulator that affects a wide range of signaling, there are various possibilities as to why high c-Myc activity is required for Bmi-1 degradation.

How does Bmi-1 contribute to stemness in HOS cells that harbor *CDKN2A* c.1_471del471? Bmi-1 has been reported to cause carcinogenesis in an Ink4a–Arf-independent manner in various cancer types (18, 23, 41, 42, 43). In ovarian cancer, suppression of Bmi-1 induces autophagy through ATP depletion (18). In hepatocarcinoma, Bmi-1 drives carcinogenesis by repressing the transforming growth factor-β–Smad signaling (44). However, GSEA showed SET KD promoted autophagy, which may be due to the inhibition of mTORC1 signaling, and the suppression of transforming growth factor-β signaling in HOS cells. Therefore, these signals do not contribute to the SET–Akt–Bmi-1 axis–mediated tumor promotion. The Ink4a–Arf1-independent cancer-promoting mechanism of Bmi-1 has not been fully elucidated and is beyond the scope of this study.

## Experimental procedures

### Cell culture

Human osteosarcoma cell line HOS, Saos-2 and U2OS, human Ewing sarcoma cell line A673, human fetal kidney epithelial cell line 293T, human colon cancer cell line SW620 and HT-29, human neuroblastoma cell line SH-SY5Y, human lung cancer cell line A549, and human pancreatic cancer cell line MIA PaCa-2 were obtained from RIKEN BRC and JCRB Cell Bank. U2OS, A673, 293T, SW620, HT-29, SH-SY5Y, A549, and MIA PaCa-2 were cultured in Dulbecco’s modified Eagle's medium supplemented with 10% fetal bovine serum and 1× antibiotic/antimycotic solution (Life Technologies). HOS and Saos-2 cells were cultured in RPMI1640 supplemented with 10% fetal bovine serum and 1× antibiotic–antimycotic solution. Contamination of mycoplasma was tested using MycoAlert (Lonza), and cells were occasionally treated with plasmocin (nacalai tesque) to prevent mycoplasma contamination.

### Plasmid, transfection, and virus production

pLVmCherry for shNT and shSET were previously generated (4). The sequences of shRNA are as follows: shNT, 5′-CAACAAGATGAGAGCACCA-3′, shSET#1, 5′-GGATGAAGGTGAAGAAGAT-3′, and shSET#2 5′-GATGAAGAGGCACTGCATT-3′. shRNA complementary DNA strands for Bmi-1 (shBmi-1: 5′-AGAGTTCGACCTACTTGTA-3′) with flanking sequences were annealed and ligated into the MluI–ClaI sites of pLVmCherry. DOX-inducible shRNA-expressing pTRIPZ plasmids were purchased from Horizon (control shRNA: RHS4743; shSET: RHS4696-201903403 and RHS4696-201904577). pLVSIN nFLAG3 human Bmi-1 and pLVSIN cFLAG3 human myr-Akt were prepared by amplifying DNA of the target gene by PCR and incorporating it into a vector using the InFusion HD Cloning Kit (Takara Bio).

To produce lentiviruses, pLV and pTRIPZ plasmids, a packaging plasmid (psPAX2), and a coat protein plasmid expressing vesicular stomatitis virus G protein (pMD2.G) were diluted in 333 μl of Opti-MEM, containing 2.5 μl of Polyethylenimine MAX (Polysciences). The mixture was added to Lenti-X 293T cells (Takara Bio) cultured in 6-well plates, incubated for 8 h, and replaced with 1.5 ml of medium. After 48 h, viral supernatants were filtered using a 2.2 μm filter (Millipore) and added to cells.

### Immunoblotting

Immunoblotting was performed as previously described (45). In brief, cells were lysed in a buffer containing 50 mM Tris–HCl (pH 8.0), 5 mM EDTA, 5 mM EGTA, 1% Triton X-100, 1 mM Na_3_VO4_4_, 20 mM sodium pyrophosphate, and Roche Complete protease inhibitor mixture. DC protein assay kit (Bio-Rad) was used to determine the protein concentration. After boiled at 100 °C for 5 min, equal amounts of proteins were applied to SDS-PAGE. Proteins were transferred onto a nitrocellulose membrane (Wako), and 0.5% skim milk in Tris-buffered saline with Tween-20 was used for blocking. After the treatment with primary/secondary antibodies, membranes were treated with ECL Pro (PerkinElmer), and immunoreactive bands were visualized using Amersham ImageQuant800 (Cytiva) or LuminoGraph II EM (ATTO). ImageJ (National Institutes of Health) was used to quantify band densities. Valosin-containing protein (p97/VCP) was used as a loading control.

Antibodies were obtained from the indicated supplier: anti-SET (Santa Cruz; catalog no.: sc-133138), anti-p97/VCP (GeneTeX; catalog no.: GTX113030), anti-c-Myc (Cell Signaling; catalog no.: 185835605), anti-E2F1 (abcam; catalog no.: ab179445), anti-Akt (Cell Signaling; catalog no.: 2920), anti–phospho-Ser473 Akt (Cell Signaling; catalog no.: 4060), anti–phospho-Thr308 Akt (Cell Signaling; catalog no.: 13038), anti-p70S6K (Santa Cruz; catalog no.: sc-230), anti–phospho-Thr389 p70S6K (Cell Signaling; catalog no.: 3001), anti-Bmi-1 (Novus; catalog no.: NBP1-96140), anti-FLAG (Sigma; catalog no.: F7425), anti-Ring1B (MBL; catalog no.: D139-3), anti-4EBP1 (Cell Signaling; catalog no.: 9452), anti–phospho-Thr37/46 (Cell Signaling; catalog no.: 2855), anti–3-phosphoinositide-dependent protein kinase-1 (Cell Signaling; catalog no.: 3062), and anti–phospho-Ser241 (Cell Signaling; catalog no.: 3438).

### Real-time PCR

About 3.0 × 10^5^ cells were seeded on 6-well plates. The next day, TRIzol Reagent (Invitrogen) was used to extract total RNA. About 0.5 μg of RNA was reverse transcribed using QuantiTect Reverse Transcription Kit (QIAGEN). The resulting complementary DNA was subjected to quantitative PCR using QuantiTect SYBRGreenI Kit (QIAGEN) and LightCycler (Roche).

The primers used were as follows: *ACTB* (Takara Bio; catalog no.: HA067803), *GAPDH* (Takara Bio; catalog no.: HA067812), and *BMI1* (Takara Bio; catalog no.: HA152363). *BMI1* mRNA levels were normalized by mRNA levels of *ACTB* or *GAPDH*. The relative quantitative value was expressed as the comparative Ct (2–[ΔCt-Cc]) method.

### Colony-formation assay

About 1.0 × 10^2^ or 1.0 × 10^3^ cells were seeded on 6-well plates. The next day, reagents were added and cultured for another 7 days. Colonies were stained with Giemsa or Gram solution. The number of colonies greater than 1 mm in diameter was counted. The area of colonies was measured by ImageJ.

### Spheroid-formation assay

About 1.0 or 3.0 × 10^3^ cells were seeded in PrimeSurface plate 24F (FUJIFILM) and cultured for 4 days. The cells were collected and treated with trypsin overnight. The number of cells was counted using a hemocytometer.

### Cell proliferation assay

About 1.0 × 10^4^ cells were seeded on 24-well plates. The cell proliferation was analyzed after 4 days using a Cell Counting Kit 8 (Dojindo) according to the manufacturer’s instructions.

### Immunofluorescence staining

About 2.5 × 10^5^ cells were seeded on 6-well plates and treated with DOX (500 ng/ml). After 2 days, 2.5 × 10^5^ cells were reseeded on the glass plate. The next day, cells were washed with PBS and fixed with 4% paraformaldehyde for 20 min at room temperature. The cells were permeabilized with 0.05% Triton X-100 in PBS for 5 min and treated with a blocking buffer (PBS containing 0.2% gelatin) for 20 min at room temperature. Rabbit anti-mTOR (1:400 dilution; Cell Signaling Technology) and mouse anti-Lamp2 (1:400 dilution; Santa Cruz [H4A3]) were treated as primary antibodies, and anti-rabbit Alexa-488 (1:200 dilution; Invitrogen) and antimouse Alexa-568 (1:200 dilution; Invitrogen) were treated as secondary antibodies. Glass plates were mounted using ProLong Gold with 4′,6-diamidino-2-phenylindole (Thermo Fisher Scientific) and observed using the LSM 710 confocal microscope (Zeiss).

### Transcriptome analysis

TRIzol Reagent was used to extract total RNA from HOS cells treated with or without DOX (500 ng/ml) for 2 days. RNA sequencing was performed by Bioengineering Lab Co. Briefly, concentrations of total RNA were determined using the Quantus Fluorometer and the QuantiFluor RNA system (Promega). The Fragment Analyzer System and Agilent HS RNA Kit (Agilent Technologies) were used to confirm quality. Libraries were prepared according to the manual using the MGIEasy RNA Directional Library Prep Set (MGI Tech Co Ltd), and the concentration of the prepared libraries was measured using the Synergy LX and QuantiFluor dsDNA System. Fragment Analyzer and dsDNA Reagent Kit (Advanced Analytical Technologies) were used to check the quality of the generated libraries. DNA nanoballs (DNBs) were prepared according to the manual using the DNBSEQ G400 RS High-Throughput Sequencing Kit (MGI Tech). The DNBs were sequenced using the DNBSEQ G400 (MGI Tech) at 2 × 100 bp. Obtained data were separated for each sample using unique barcode combinations to generate FASTQ files. Raw microarray data were deposited in Gene Expression Omnibus as accession number GSE235621. The obtained next-generation sequencing data were subjected to reads cleaning with TrimGalore (version 0.6.6). After a quality control step, the filtered short reads were mapped to the reference genome (hg38) with HISAT2 (version 2.2.1). The strand-specific counts of fragments from each sample were normalized with the transcripts per million method using StringTie (version 2.1.5). Count data are shown in supporting information as gene_count_matrix.csv.

For the genes with an average count of 10 or more, GSEA (version 4.2.2) (46) was carried out between datasets of HOS cells expressing shNT and shSET induced by DOX treatment. The normalized enrichment score and NOM *p* value were shown on enrichment plots. For the enrichment analysis using Enrichr (47), TCC-GUI (48) was used to extract differentially expressed genes setting the cutoffs for fold change and *p* value at −1.5 to +1.5 and 0.01, respectively.

### Statistical analysis

The results of the bar graphs were shown as mean ± standard deviation. Student’s *t* test was used for comparisons between two groups, and one-way ANOVA followed by Fisher’s least significant difference test was performed for comparisons between three or more groups. For all analyses, statistical significance was set at *p* < 0.05.

## Data availability

All data are contained within the article and the supporting information.

## Supporting information

This article contains supporting information.

## Conflict of interest

The authors declare that they have no conflicts of interest with the contents of this article.
