# Supplementary material for: PP2A inhibitor SET promotes mTORC1 and Bmi1 signaling through Akt activation and maintains the colony-formation ability of cancer cells
Source: J Biol Chem. 2023 Dec 22;300(1):105584. doi: 10.1016/j.jbc.2023.105584 (PMC10826185; doi:10.1016/j.jbc.2023.105584)
Supplement: Supporting Figures S1–S7 [file mmc1.docx]

# **Supplemental Figures**

## Supplemental Fig. 1

(A−D) HOS cells were stably expressed shNT and shSET. (A−B) Protein levels were analyzed by immunoblotting. Representative images (A) and quantitative data (B) are shown. The band densities were normalized to shNT at 100%. (C−D) A colony formation assay. Representative pictures (C) and quantitative data (D) are shown. The number of colonies was normalized to shNT at 100%. (E-M) SW620 cells were stably expressed shNT and shSET. Protein levels (E−F) and phosphorylation levels of c-Myc at Ser62 (I−J) were analyzed by immunoblotting. Representative images (E, I) and quantitative data (F, J) are shown. The band densities were normalized to shNT at 100%. (G−H) A colony formation assay. Representative pictures (G) and quantitative data (H) are shown. The number of colonies was normalized to shNT at 100%. (K) MYC mRNA levels were analyzed by real-time PCR. (L−M) Cyclophosphamide (CHX) chase assay. Representative images (L) and quantitative data (M) are shown. (N−O) A colony formation assay for HOS cells expressing DOX-inducible shNT and shSET without DOX. Representative pictures (N) and quantitative data (O) are shown. The number of colonies was normalized to shNT at 100%. (P−S) HOS cells were expressed shNT and shSET #2 by DOX treatment. (P−Q) Protein levels were analyzed by immunoblotting. Representative images (P) and quantitative data (Q) are shown. The band densities of shSET-expressing cells were normalized to those of shNT-expressing cells as 100%. (R−S) A colony formation assay. Representative pictures (R) and quantitative data (S) are shown. The number of colonies of shSET was normalized to those of shNT at 100%. *: *P*<0.05. NS: Not significant different.

## Supplemental Fig. 2

HOS cells were expressed shNT and shSET by DOX treatment. (A–B) Gene set enrichment analysis (GSEA) was performed for the transcriptome data. (A) Top 5 gene sets from the results of GSEA HALLMARK. (B) Enrichment plots for MTOR_UP.N4.V1_DN in C2. (C−D) HOS cells were expressed shNT and shSET #2 by DOX treatment. Protein levels were analyzed by immunoblotting. Representative images (C) and quantitative data (D) are shown. The band densities of shSET #2-expressing cells were normalized to those of shNT-expressing cells at 100%. (E−F) HOS cells were expressed shNT and shSET by DOX treatment. Immunoblotting was performed for indicated antibodies. Representative images (E) and quantitative data (F) for phospho-Thr37/46 4EBP1 and phospho-Ser241 PDK1 are shown. The band densities were normalized to shNT at 100%. (G) Immunofluorescent staining for mTOR (green), Lamp2 (red), and DAPI (blue). Representative images from two independent experiments are shown. Scale bar: 10 μm. (H) Quantitative data of Fig. 2G. *: *P*<0.05. NS: not significant different.

## Supplemental Fig. 3

(A−C) The effect of Akt inhibitor VIII (A), rapamycin (B), and torin1 (C) on Akt or p70S6K phosphorylation were analyzed. Representative images from two independent experiments are shown.

(D−E) The effects of torin1 on the colony formation ability of HOS cells were analyzed. Representative pictures (D) and quantitative data (E) are shown. The total area of colonies was normalized to untreated cells at 100%. (F–H) HOS cells were expressed shNT and shSET by DOX treatment. (F) The effect of the myr-Akt expression on p70S6K phosphorylation was analyzed. Representative images from two independent experiments are shown. (G−H) A spheroid formation assay. Representative images (G) and quantitative data (H) are shown. The number of cells was normalized to shNT/mock at 100%. *: *P*<0.05. Scale bar: 100 μm.

## Supplemental Fig. 4

(A) The volcano plot for the transcriptome analysis of shNT and shSET expressing HOS cells. (B−C) HOS cells were expressed shNT and shSET #2 by DOX treatment. Protein levels were analyzed by immunoblotting. Representative images (B) and quantitative data (C) are shown. The band densities of shSET-expressing cells were normalized to those of shNT-expressing cells at 100%. (D) HOS cells were treated with okadaic acid (OA, 100 nM) for two hours and immunoblotting was performed for indicated antibodies. (E−F) Quantitative data of Fig. 4J. (G−H) HOS cells were expressed shNT and shSET by DOX treatment. Protein levels were analyzed by immunoblotting. Representative images (G) and quantitative data (H) are shown. The band densities of shSET were normalized to shNT at 100%. (I−J) The effect of Akt inhibitor VIII (10 μM, 24 h) on RING1B protein expressions was analyzed. Representative images (I) and quantitative data (J) are shown. The band densities were normalized to untreated cells at 100%. *: *P*<0.05. (K−O) HOS cells were expressed shNT and shSET by DOX treatment and were treated with rapamycin (1 μM, 24 h). Protein levels were analyzed by immunoblotting. Representative images (K) and quantitative data (L–O) for the indicated antibodies are shown. The band densities were normalized to shNT/untreated at 100%.

## Supplemental Fig. 5

(A−D) HOS cells were treated with PTC-209 (0.4 μM, 24 h), and the effect on protein expression (A−B) and colony formation ability (C−D) were analyzed. Representative images of immunoblotting (A) and quantitative data (B) are shown. The band densities were normalized to untreated at 100%. Representative pictures of colony formation assay (C) and quantitative data (D) are shown. The number of colonies was normalized to shNT at 100%. (E−F) Colony formation assay. HOS cells were expressed shNT and shSET by DOX treatment and were treated with PTC-209. Representative pictures (E) and quantitative data (F) are shown. The number of colonies was normalized to shNT/ untreated at 100%. (G–H) HOS cells were expressed shNT and shSET by DOX treatment. The effect of the FLAG-Bmi-1 on spheroid formation was analyzed. Representative images (G) and quantitative data (H) are shown. The number of cells was normalized to shNT/mock at 100%. *: *P*<0.05. *: *P*<0.05. Scale bar: 100 μm.

##

## Supplemental Fig. 6

(A–B) Immunoblotting was performed for the indicated antibodies. Representative images (A) and quantitative data (B) from three or more independent images are shown. The band densities were normalized to those of HOS cells at 100%. (C−D) Cells were expressed shNT and shSET and immunoblotting was performed for the indicated antibodies. Representative images (C) and quantitative data (D) from three or more independent images are shown. (E–I) A549 cells were expressed shNT and shSET and the effects of FLAG-Bmi-1 on colony formation (F–G) and spheroid formation (H–I) abilities were analyzed. Immunoblotting was performed to confirm SET KD and Bmi-1 expression (E). Representative images (F, H) and quantitative data (G, I) were shown. The total area of colonies and the number of cells were normalized to shNT/mock at 100%. Scale bar: 100 μm. (J–N) A549 cells were expressed shNT and shSET and the effects of myr-Akt on colony formation (K–L) and spheroid formation (M–N) abilities were analyzed. Immunoblotting was performed to confirm SET KD and myr-Akt expression (J). Representative images (K, M) and quantitative data (L, N) were shown. The total area of colonies and the number of cells were normalized to shNT/mock at 100%. *: *P*<0.05. Scale bar: 100 μm.

Supplemental Fig. 7

(A−C) Transcriptome data of indicated cell lines were obtained from the depmap portal. (A) Heatmap hierarchical clustering displays the expression of genes associated with sensitivity for SET KD-mediated Bmi-1 degradation. (B) Gene set enrichment analysis for the transcriptome data of cell lines sensitive and insensitive for SET KD-mediated Bmi-1 degradation. Enrichment plots for MYC_TARGETS_V1 are shown. (C) Quantitative data of Fig. 6E. NS: Not significant different.
